# Nutraceutical and Medicinal Importance of Marine Molluscs

**DOI:** 10.3390/md22050201

**Published:** 2024-04-27

**Authors:** Yvan Anderson Tchangoue Ngandjui, Tsotlhe Trinity Kereeditse, Ilunga Kamika, Lawrence Mzukisi Madikizela, Titus Alfred Makudali Msagati

**Affiliations:** Institute for Nanotechnology and Water Sustainability, College of Engineering, Science and Technology, University of South Africa, Florida Science Campus, Johannesburg 1705, South Africa; 21123187@mylife.unisa.ac.za (T.T.K.); kamiki@unisa.ac.za (I.K.); madiklm@unisa.ac.za (L.M.M.)

**Keywords:** marine molluscs, chemical ecology, extraction techniques, nutraceutical importance, biological properties, bioactive compounds

## Abstract

Marine molluscs are of enormous scientific interest due to their astonishing diversity in terms of their size, shape, habitat, behaviour, and ecological roles. The phylum Mollusca is the second most common animal phylum, with 100,000 to 200,000 species, and marine molluscs are among the most notable class of marine organisms. This work aimed to show the importance of marine molluscs as a potential source of nutraceuticals as well as natural medicinal drugs. In this review, the main classes of marine molluscs, their chemical ecology, and the different techniques used for the extraction of bioactive compounds have been presented. We pointed out their nutraceutical importance such as their proteins, peptides, polysaccharides, lipids, polyphenolic compounds pigments, marine enzymes, minerals, and vitamins. Their pharmacological activities include antimicrobial, anticancer, antioxidant, anti-inflammatory, and analgesic activities. Moreover, certain molluscs like abalones and mussels contain unique compounds with potential medicinal applications, ranging from wound healing to anti-cancer effects. Understanding the nutritional and therapeutic value of marine molluscs highlights their significance in both pharmaceutical and dietary realms, paving the way for further research and utilization in human health.

## 1. Introduction

The size of the oceans and the rich biodiversity of the organisms living there make the marine environment an ideal source for bioactive compounds [1]. Oceans make up more than 70% of the Earth’s surface and have more than 200,000 species of animal and plant life [2]. As a result, there may be a great chance to discover new compounds in the maritime environment [3,4,5]. Marine ecosystems are incredibly complicated, with pressure restrictions varying from 1 to 1000 atm, nutrient limits (oligotrophic or eutrophic), and thermal parameters ranging from 0 °C in the Antarctic to 350 °C in deep hydrothermal areas [6]. To endure the high stressors present in marine environments, marine species developed chemically or structurally unique bioactive secondary metabolites as part of their various biosynthetic pathways. These secondary metabolites were believed to be caused by a variety of chemically mediated interactions that have been studied, such as interactions between planktonic organisms and predators–prey and seaweed–herbivores, as well as chemical defences against pathogenic marine microbes and fouling organisms [7,8]. Among the marine organisms that inhabit the marine ecosystem are the marine molluscs from the phyla Mollusca which fall under marine invertebrate species [9]. Molluscs are among the class of marine organisms that make up 7% of all marine animals, and the phylum Mollusca is the second most common animal phylum, with 100,000 to 200,000 species, of which more than 52,000 have been found and classified [10]. This indicates that Mollusca offers a broad spectrum of organisms [11,12]. 

In the search for bioactive metabolites, the scientific world has paid phylum Mollusca significant attention. Soft-bodied molluscs without shells are the most frequently chosen for natural product isolation because it is thought that they will be a rich source of protective secondary metabolites [10,13,14]. Due to their sessile or slow-moving nature and lack of a protective covering, which leaves them exposed to predators, they must develop chemical defences [10,13,15]. The shell-covered molluscs must stretch their muscular foot outside into a hostile environment full of predators and microbial infections to feed and move [16,17,18]. The quantity of compounds isolated from molluscs and the growing body of research on molluscs, however, do not correspond to the diversity of the available species. In fact, less than 1% of molluscan species has undergone chemical analysis, as numerous studies have examined the same species in various ecological environments [10]. The importance of marine molluscs as alternatives to traditional fisheries has significantly increased during the past several decades in many different parts of the world [19]. As a result, the development of potential therapeutic leads, functional foods, nutraceuticals, and pharmaceutical products benefited greatly from coastal ecosystems [20]. The present review article extensively comprehended the importance of marine molluscs as a potential source of nutraceuticals as well as natural medicinal drugs. Future research endeavours, particularly concerning the development of functional foods, nutraceuticals, and therapeutic leads with multiple bioactivities, might benefit from this comprehensive review of the molluscan phylum owing to the diverse number of species.

## 2. Definition and Types of Marine Molluscs

**Figure 1:**
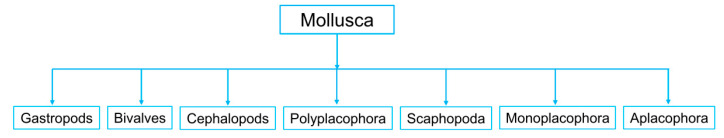
Classes of marine molluscs.

A broad group of invertebrate organisms that make up the phylum Mollusca are known as marine molluscs, or simply molluscs [21]. They can be found in a variety of marine settings, including oceans, seas, and estuaries, and they play a variety of ecological roles in marine ecosystems [22,23]. Molluscs are distinguished by their soft bodies, which are frequently shielded by hard shells; however, not all molluscs have shells [21,24]. Snails, clams, squids, and octopuses are among the well-known and significant creatures that belong to this phylum [20,25,26]. Based on their anatomical characteristics and ecological functions, molluscs can be roughly divided into seven main groups as mentioned in Figure 1 below, with each having distinct characteristics and adaptations [27]. 

Molluscs contribute to biodiversity and the dynamics of marine environments by acting as both predators and prey, and their largest group is called the *Gastropoda* [28]. Most *Gastropod* species have a single, coiling shell, but some do not. They can be found in various maritime locations and have different feeding habits [29]. They walk on a muscular foot, and their radula (a feeding organ) is frequently well-developed [30,31]. Snails, sea slugs, and nudibranchs are a few examples [32]. In the *Bivalvia* class, the two-part, hinged shell of *Bivalves* is what distinguishes them from other animals; as examples, we have clams, mussels, oysters, and scallops [33]. *Bivalves* are filter feeders that draw food particles from the water with the help of their gills [34,35]. The third largest group of molluscs, called *Cephalopoda,* includes some of the smartest and most active individuals [36,37]. They are recognized for their sophisticated behaviours and intricate nerve systems and are carnivorous [38]. They possess a distinct head, a shrunken or internalized shell (or, in rare cases, no shell at all), and a group of strong tentacles that are fitted with suckers [39]. Squids, octopuses, nautiluses, and cuttlefish are other examples [40,41].

The molluscs in the *Polyplacophora* class are also known as chitons, and polyplacophorans have an oval body shape with eight overlapping armoured plates covering it [42]. They are mostly found in intertidal areas, and their unique feeding organ, the radula, is used to scrape algae off rocks [43]. The molluscs belonging to the *Scaphopoda* class are often known as "tusk shells" because of their long and tubular shells [44]. They are organisms that live in soft sediments and burrow, feeding on debris or tiny particles [45]. The *Monoplacophora* class is scarce and those that are extinct have been discovered to be characterized by segmented bodies and a single, cap-like shell [46]. Before the discovery of living specimens in deep water, they were believed to be extinct [47]. Another less well-known molluscan group called *Aplacophorans* consists of tiny, worm-like organisms that lack a shell or have scaled-down, spicule-like features [15]. They frequent locations in deep water and consume tiny invertebrates [48]. They can be classified as *Caudofoveates* and *Solenogasters* [10,49,50].

**Figure 2:**
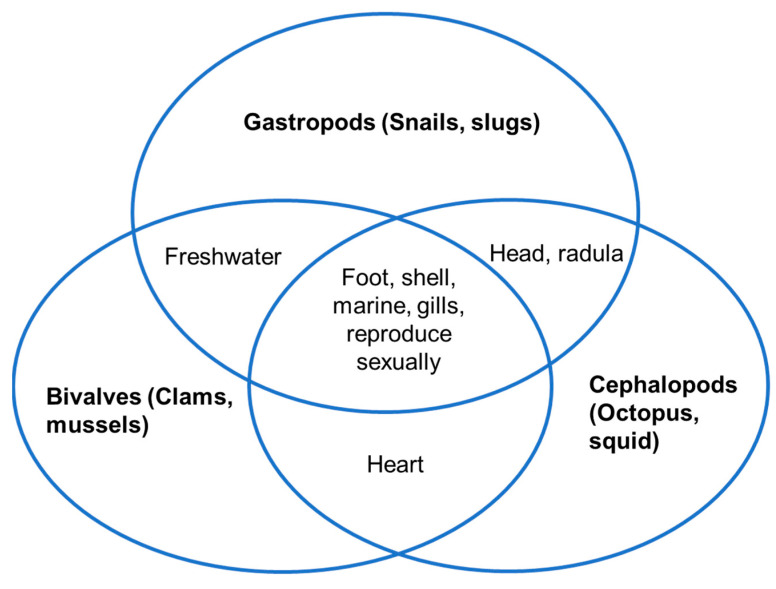
Major classes of living molluscs and their characteristics.

Among all these classes of molluscs, the three that can be classified as the major classes are *viz Gastropods*, *Bivalves,* and *Cephalopods*, and we can find their main similarities in Figure 2, below [10].

## 3. Marine Molluscs and Chemical Ecology

Chemical compounds are frequently used by various marine organisms including marine molluscs for communication, self-defence from predators and pathogens, attracting mates, attacking prey, and competing for resources [51,52,53]. Chemical defences are a common strategy used by marine molluscs to ward off predators [54]. To avoid being consumed, they may discharge toxic substances like toxins or ink [55]. For instance, cone snails are renowned for injecting their prey with powerful venom by utilizing specialized radula (a feeding organ) [56]. The vibrant sea slugs known as nudibranchs can retain the toxic compounds from their food, such as sponges or cnidarians, and employ them for self-defence. Certain compounds, including *kahalalide F*, have proven to be predator-defensive in *sacoglossan* prey [57]. Investigations into Antarctic animals have also shown how natural compounds can fend off predators [58]. They store these toxins in their bodies to act as a deterrence to possible predators [55]. Molluscs emit chemical cues that can either draw in or drive away predators and prey [27]. For instance, certain predatory molluscs use chemical cues emitted by their prey to identify them, whereas possible prey species may release chemicals to warn other creatures of the presence of predators, causing them to take protective measures [27,59].

In marine chemical ecology, the feeding stimulants and compounds that entice consumers to food sources are significant yet understudied. Certain compounds, including lipids, carbohydrates, and amino acids, can draw or keep away marine *Gastropods* [60]. Crabs and oysters use scent plumes to find and detect their bivalve prey [61,62,63]. It is believed that because there are more consumers in tropical areas, these organisms have greater defences against them [64]. Secondary metabolites may frequently act as consumer defences, according to geographic trends in their prevalence [65,66]. Secondary metabolites frequently exhibit intraspecific variation that can differ within and between individuals, groups, and geographical areas. Intraspecific diversity in chemical defences may result from induction brought on by consumer attack or physical stress [67]. Studying various prey features and their integration is crucial for understanding chemical defences because a prey’s nutritional quality and chemical defences interact to impact that prey’s susceptibility to consumers [68].

Their nutritional sources have an impact on the chemical diversity of molluscs as well [69]. According to research, in addition to molluscs being able to biosynthesize compounds de novo, they also bioaccumulate them from their diets (especially algal diets) or bioaccumulate then sequester (chemically modify) them [70,71]. Both *Gastropods* and *Bivalves* contain all known secondary metabolites, although due to their involvement in reproduction, terpenes predominate in gastropods whereas sterols are more common in bivalves [72]. Both types contain alkaloids and polyproprionates, although aliphatic nitrogenous chemicals are less frequent. Analogs, which share a similar structure, are frequently discovered in small groups and in several locations for the same species [10]. The sea hare *Aplysia kurodai*, for instance, produced 25 different compounds, including terpenes, nitrogenous aliphatic compounds, macrolides, and fatty acid derivatives. *Aplysia dactylomela*, a similar species, generated 58 chemicals, mostly terpenes, which they probably got from their algal meals [10]. This explains why related species—especially those in the same family but inhabiting diverse habitats—share similar feeding metabolites and biosynthesized substances [69].

Molluscs’ survival, reproduction, growth, and adaption methods are ultimately shaped by the composition of their lipids, especially their fatty acids, which are affected by elements like nutrition, metabolic processes, ambient conditions, and reproductive cycles [69]. Different species of molluscs have different feeding behaviours, such as filter feeding or eating detritus, which causes variations in their fatty acid profiles [73]. Specific fatty acids, including C20:5: ω3 from diatoms and C22:6: ω3 from dinoflagellates, are found in diets high in dinoflagellates and diatoms, and they help to make up the fatty acid profile of molluscs [74,75]. A major environmental component influencing fatty acid profiles is temperature, with greater summertime temperatures causing more lipid build-up, especially of polyunsaturated fatty acids (PUFAs) [76,77]. This build-up most likely has something to do with preserving the melting point of cellular lipids under changing circumstances [77]. Fatty acid levels and patterns are also significantly influenced by metabolic processes, especially reproductive cycles [78]. There may be a link between reproductive cycles and fatty acid profiles since reproductive processes require a significant amount of energy, primarily in the form of fatty acids [79]. Additionally, high energy levels are needed for growth processes, and the requirements for fatty acids vary depending on the organ [80,81].

It is worth noting that molluscan chemicals are produced in response to environmental conditions like temperature, salinity, and seasonal changes. Chemical components may differ because of changes to these parameters [69]. For instance, certain temperature and salinity conditions have an impact on fatty acids and amino acids. The composition of chemicals in molluscs is ultimately influenced by environmental and biological factors, including food availability and metabolic activity [69].

## 4. Extraction Techniques of Bioactive Compounds from Marine Molluscs

Marine molluscs are known to contain various bioactive compounds (secondary metabolites in different structural classes: terpenes, polypropionates, those that are nitrogenous (aromatic), those that are nitrogenous (aliphatic), polypeptides, macrolides, fatty acid derivatives, sterols, miscellaneous) with potential health benefits [10,82]. Bioactive molecules are ‘concealed’ in the primary structures of the tissue samples of molluscs which are released by mechanical and chemical processes [83]. Extracting these compounds involves a series of techniques to successfully isolate and concentrate the desired components. Several extraction techniques have been developed to isolate bioactive compounds according to their chemical characterization, such as proteins/amino acids, carbohydrates, and lipids [84]. It is standard procedure to remove non-functional components from an isolate to make it amenable for analysis. The right choice of solvents, buffers, pH ranges, temperatures, etc., will produce optimal conditions for the extraction of the desired compounds [85]. With a few small variations, the extraction methods for bioactive chemicals, whether they be crude or refined isolates, are fundamentally the same. Additional approaches for isolating compounds from mixtures include centrifugation and filtering. These isolates are concentrated by drying. The primary variables in the isolation of a targeted compound and its inherent bioactivities are the solvents and extraction conditions [86]. Therefore, extraction techniques influence the structure, composition, and beneficial properties (mechanism of action) of mollusc-derived extracts, so it is important to match the extraction method with the desired outcome (target compounds) as well as molluscs species to obtain the specific isolate [83]. These techniques will also differ according to the extraction yield and time, the reproducibility, the volume of organic solvents used, and the co-extraction of other compounds [87]. We can then classify the common extraction techniques used for the extraction of active compounds from marine molluscs into two general categories which are conventional and non-conventional. 

### 4.1. Conventional Techniques

These techniques, also known as traditional extractions or solid–liquid extractions, are the most frequently and commonly used extraction techniques [88]. This traditional extraction can be done in numerous ways, viz, boiling the sample and solvent with or without stirring for a certain duration, refluxing using soxhlet, percolation, and maceration with continuous stirring [89]. In these extractions, several solvents at high volumes are used including water, methanol, ethanol, acetonitrile, ethyl acetate, acetone, and dichloromethane, based on the compounds of interest [90]. It is important to mention that these techniques are manual operations that may require more time to extract depending on the solvent diffusion rate and time and there is a high chance of the degradation of thermolabile compounds for temperature-dependent methods like soxhlet [86,91]. Due to practicality, economic, environmental, and energy concerns, scaling up these technologies to an industrial scale would also be challenging [92]. Thus, several recently developed extraction methods were presented to address the methods’ shortcomings [93]. 

### 4.2. Non-Conventional Techniques

The growing recognition of the need for cost-effective and ecologically friendly methods has led to the development of a variety of alternative techniques. New non-traditional extraction techniques, such as enzyme-assisted extraction (EAE), microwave-assisted extraction (MAE), pressurized liquid extraction (PLE), subcritical water extraction (SWE), supercritical fluid extraction (SC-CO_2_), and ultrasound-assisted extraction (UAE) have been developed to overcome the drawbacks of conventional methods [85]. These methods, which will be discussed below, produce high yields of better quality for the recovery of bioactive chemicals and are quick, non-toxic, and affordable [85]. The advantages and disadvantages of these modern extraction methods are shown in Table 1.

#### 4.2.1. Enzyme-Assisted Extraction (EAE)

Enzyme-assisted extraction (EAE) has several benefits compared to traditional extraction techniques, such as a lower operating temperature and the use of environmentally acceptable solvents. Although EAE is not a brand-new technique for extracting bioactives from marine organisms, it is a developing field of study due to ongoing efforts to optimize and intensify the extraction process and find new reliable enzymes [94]. The EAE method involves a catalytic hydrolysis reaction to disrupt the cell wall under optimal experimental conditions and release intracellular components into the extraction medium [95]. Proteases and carbohydrases, which are food-grade digestive enzymes, can be employed to macerate tissues and disintegrate natural matrices’ cell walls to release the contents of the cells. Additionally, important factors to consider include the temperature, pH, the ratio of substrate to enzyme, the kind of solvent (water or a buffer with the right pH), and agitation [95]. The EAE has been utilized in extracting fatty acids (C13:0, C14:0, C16:0, C16:1ω7, C16:2ω6, C18:1ω9, C18:2ω5, C18:2ω6, C18:4ω3, C20:1ω7, C20:1ω9, C20:5ω5, C22:6ω3) from the molluscan species *Patinopecten yessoensis Jay* using papain enzyme [96]. The yields recovered were 23.7%, 19.5%, and 55,4% for saturated fatty acids (SFA), monounsaturated fatty acids (MUFA), and polyunsaturated fatty acids (PUFA), respectively [96].

#### 4.2.2. Microwave-Assisted Extraction (MAE)

An efficient extraction method called microwave-assisted extraction (MAE) makes use of microwave radiation to quickly remove various chemicals from natural sources. MAE is a straightforward method with a wide range of uses that uses little organic solvent and few molecules. An oscillating electric field with frequencies between 300 MHz and 300GHz or a wavelength in MAE between 1mm and 1m is used in this approach. These frequencies induce polar molecules to oscillate, which results in friction between and within molecules. The collision of these frictions and ion charges results in rapid heating. Increased heating leads to the collapse of the membrane and cell wall through pressure. When the pressure inside the sample cells rises, the chemicals move into the extraction solvent more quickly [85]. Polar solvents are preferable to non-polar solvents when using the MAE approach because they have larger dielectric strengths, which allow them to absorb more energy and raise the solvent’s temperature faster. Water, methanol, ethanol, acetone, ethyl acetate, and hexane are the solvents with decreasing dielectric constants [97]. This method was employed in the extraction and determination of four widely used antidepressants (venlafaxine, citalopram, sertraline, and fluoxetine) and two metabolites (o-desmethylvenlafaxine and norsetraline) from marine organism species (fish, echinoderms, molluscs, and algae). Three main antidepressants were detected, but only two could be quantified: citalopram: 5.83 ng g^−1^ and sertraline: 6.58 ng g^−1^ [98].

#### 4.2.3. Subcritical Water Extraction (SWE)

Subcritical water extraction (SWE) is a green extraction method that produces excellent yields in a short amount of time while using less organic solvent. SWE is a system that is good for the extraction of substances from biologically active compounds [99]. To preserve the liquid condition, this method uses water at high pressures and temperatures (100–374 °C). SWE differs from conventional extraction methods due to several characteristics of water, including its strong polarity, high dielectric and high boiling point relative to its mass [100]. The SWE approach is predicated on the idea that higher temperatures alter the properties of water. In the SWE process, a temperature increase causes the permittivity to drop. This increases the rate of diffusion and lowers the water’s surface tension and viscosity. This improves the mass transfer of the water, which raises the yield and extraction rate. Another characteristic that changes during subcritical water extraction is the dielectric constant. In its native liquid state, water has a high dielectric constant and is strongly polar. The dielectric constant of water decreases to levels like those of organic solvents when it reaches a subcritical temperature. For bioactive compounds with lower polarity, especially those in the medium polarity range, lowering the dielectric constant enhances selectivity [99,100]. The SWE technique has been employed in extracting bioactive compounds from *Haliotis iris*, where the effect of subcritical water temperature (110–280 °C) on extraction performance was studied [101]. Temperatures between 220 and 250 °C were shown to have the maximum concentration of bioactives, glycogen and phenolic content, and antioxidant activity. The carbohydrate content peaked at 110 °C and subsequently began to break down at higher temperatures; moreover, when the temperature rose over 160 °C, the amounts of protein and amino acids similarly dropped [101].

#### 4.2.4. Supercritical Fluid Extraction (SC-CO_2_)

The method of separating a component from a matrix component by utilizing supercritical fluid as an extracting solvent is known as supercritical fluid extraction. Any material at a temperature and pressure higher than the critical point is referred to as a supercritical fluid. The precise pressure and temperature above which there are no liquid or gas phases is known as the critical point [102]. The density and viscosity of the supercritical fluid (SFE) are like those of a liquid and a gas, respectively, while the fluid’s dispersion is in between the two states. SFE dissolves components as a liquid after passing through the solid as a gas. Typically, supercritical extraction uses CO_2_ to extract valuable compounds from natural sources at a high pressure. As a co-solvent, organic solvents can change the solvent polarity, allowing for a greater variety of extraction methods due to the poor solubility of supercritical CO_2_ [103]. It is cheap, tasteless, odourless, non-flammable, and non-toxic to extract supercritical CO_2_ from the water. As there is no surface tension and the viscosities are smaller than in those in liquids, dispersion happens more quickly in supercritical fluids. This makes the procedure quick because solvents may flow through tiny matrix pores that liquids cannot reach. Nutraceutical, fragrance, essential oil, food, and fuel industries are among the businesses that can greatly benefit from this technology [103]. SC-CO_2_ was used for the recovery of bioactive Tyrian purple precursors including tyrindoleninone, 6-bromoisatin, and tyriverdin from the marine mollusc *Dicathais orbita* [104]. In this study, at 15, 30, and 50 MPa CO_2_, the impact of pressure on the selective extraction of brominated indoles was examined and contrasted with the composition and yields of conventional chloroform extract. More lipophilic tyrindoleninone at 35 and 29% and tyriverdin at 23 and 40% of the extract composition were solvated by supercritical CO2 at pressures of 30 and 50 MPa, respectively, while extracts obtained from 15 MPa selectively concentrated 6-bromoisatin at 78% of the extract composition [104].

#### 4.2.5. Ultrasound-Assisted Extraction (UAE)

Ultrasound-assisted extraction is a useful non-conventional technology that offers higher product extraction yields in less time. This method is appropriate for the extraction of bioactive compounds because it can yield large amounts of the compounds in a brief time [105]. Sound waves ranging from 20 to 100 kHz are used in ultrasonic-assisted extraction. The system receives energy in direct proportion to the variation in acoustic pressure caused by passing waves, which produce zones of high and low pressure. By increasing the solvent’s penetration into materials and the surface area that encounters the liquid and solid phases, ultrasound increases the efficacy of extraction [106]. This method was used for the extraction of bioactive compounds from *Nerita albicilla* (gastropod), *Perna viridis* (mussel), and brachyuran crabs *Ozius rugulosus* [82].

**Table 1:** Advantages and disadvantages of non-conventional extraction methods.

Extraction Methods	Advantages	Disadvantages	References
Enzyme-assisted extraction (EAE)	(i) It is a non-toxic, environmentally beneficial method. (ii) It enables the production of large yields of bioactive compounds. (iii) It transforms raw materials that are insoluble in water into those that are soluble. (iv) The approach is rather inexpensive due to the utilization of food-grade enzymes.	(i) Enzyme treatment is often a lengthy process that might take hours or even days.	[107,108]
Microwave-assisted extraction (MAE)	(i) Minimal solvent usage and treatment duration. (ii) Elevated extraction yields.	(i) It is only possible to utilize solvents with strong dielectric characteristics. (ii) The most thermolabile compounds may degrade over time when using open vessels. (iii) Substantial energy use.	[109,110]
Subcritical water extraction (SWE)	(i) Use of non-toxic solvents.	(i) Expensive prices for the necessary high-pressure apparatus. (ii) Thermolabile compounds may degrade because of high-temperature extractions.	[111,112]
Supercritical fluid extraction (SC-CO2)	(i) Increased selectivity due to the ability to control a compound’s solubility in a supercritical fluid. (ii) The extraction is solvent-free as the CO2 is eliminated and leaves no trace. (iii) Ideal for extracting thermolabile compounds.	(i) Substantial expenses for the necessary high-pressure apparatus. (ii) Toxic modifiers, such as methanol, are necessary for the extraction of polar chemical compounds. (iii) May require more time than the other available methods.	[113,114]
Ultrasound-assisted extraction (UAE)	(i) Minimal solvent usage and treatment duration. (ii) A high degree of cell disruption efficiency. (iii) High extraction yields. (iv) Inexpensive.	(i) It works best with solvents with low vapor pressure, low viscosity, and low surface tension. (ii) Oversonication has the potential to degrade extract quality.	[115,116]

It is worth noting that few researchers have used non-conventional extraction techniques in the extraction of bioactive compounds from marine molluscs. However, more work needs to be done to conclude which non-conventional method is the best for extracting various groups of bioactive compounds from marine molluscan species. The optimal method will depend on the rate at which different parts of molluscan bodies dissolve by disrupting intermolecular interactions, breaking cell membranes, and releasing cellular contents.

## 5. Nutraceutical Importance of Marine Molluscs

The molluscs are classified as edible shellfish, which have been traditionally utilized as a functional food with health benefits [117]. The scientific community gave marine molluscs a lot of consideration since they play a significant role as useful ingredients for the food industry and offer a variety of advantages for human health, either directly or after processing [12]. The nutraceutical and functional food industry is currently growing in popularity throughout the world, as an alternative to the pharmaceutical industry [118]. Due to their high nutritional value, marine molluscs have been a staple food for many societies for ages [119]. They are great suppliers of important nutrients such as omega-3 fatty acids, vitamins, minerals, and proteins [120]. Out of these seven classes of molluscs, *Bivalves* (mussels, oysters, clams, and scallops), *Cephalopods* (squid, cuttlefish, and octopus), and *Gastropods* (whelks, sea snails, cockle, and abalone) represent economically considerable molluscs and they constitute common edible seafood items in human consumption which were used as a balanced protein resource [20]. Additionally, these molluscan species offer vital amino acids, which are important for both overall health and the development of muscles [121]. Therefore, the species belonging to this phylum represent rich sources of chemical diversity and health products, allowing for the evolution of nutritional supplements, and it is worth noting that the nutraceutical content of marine molluscs can vary based on factors such as their species, habitat, diet, and harvesting methods [11,12,20].

The compounds from marine molluscs that have shown beneficial health effects and potential uses in food and medical applications include proteins and peptides (collagen, gelatin, and albumins), polysaccharides (carrageenan, agar-agar, fucans, fucanoids, chitin, chitosan, and derivatives), lipids (phospholipids, sterols, and fatty acids), polyphenolic compound pigments (phlorotannins, β-carotene, chlorophylls, and lutein), enzymes (gastric proteases, pepsins, gastricsins, chymosins, serine, cysteine, lipases, and transglutaminase), and fat- and water-soluble vitamins [12,121].

### 5.1. Proteins and Peptides

Marine molluscs are rich in high-quality proteins, making them valuable sources of essential amino acids/bioactive peptides (collagen, gelatine, and albumins) with potential antioxidant, antihypertensive, and anticoagulant properties [122]. These compounds may contribute to cardiovascular health, tissue repair, immune function, and muscle growth [123]. Albumin extracted from molluscs has anticoagulant and antioxidant properties and is applied as a whipping, suspending, or stabilizing agent [124]. Gelatin extracted from giant squid tunics (*Dosidicus gigas*) demonstrated antioxidant activity after hydrolysis with trypsin, chymotrypsin, or pepsin. Bivalve (*Sepia officinalis*) protein hydrolysates and peptides possess antioxidant activity [125].

### 5.2. Polysaccharides 

Marine molluscs, such as certain types of shellfish, can be a source of various polysaccharides (carrageenan, agar-agar, fucans, fucanoids, chitin, chitosan, and derivatives) with potential applications in the food industry [126]. Polysaccharides derived from the shells of marine molluscs include chitin and chitosan, as well as their derivatives, which are used as gelling agents, edible protective films, fruit clarification, and de-acidification, and have antitumor, bactericidal, and fungicidal properties [127]. They also have increased dietary fiber contents and reduced lipid absorption [124]. The several species of chiton, molluscan species *Rapana venosa*’s eggs, and cephalopod *sepia prashadi* have chitin and chitosan which possess antioxidant, anti-microbial, anti-viral, and anti-hypertension properties [125]. *Bivalve Perna canaliculus* exhibit Glycosaminoglycans/Biolane and Glycosaminoglycans/GlycOmega-PLUS with anti-inflammatory and anti-arthritic properties, respectively [125]. 

### 5.3. Lipids 

The lipid content of molluscs is their most significant nutritional feature and lipids perform essential biological roles as signalling molecules, components of the cell membrane’s structure, and molecules that store energy [128,129]. Molluscan-derived lipids consist of molecules such as fatty acids and their derivatives (i.e., tri-, di monoglycerides, and phospholipids), as well as other sterol-containing molecules (i.e., cholesterol) [130,131]. 

Phospholipids are the most dominant lipids in marine molluscs, probably because they are one of the main lipid structural components of biological membranes [132]. Phospholipids have various health benefits including anti-inflammatory properties since they can act as lipid mediators of inflammation that can influence immunological processes at the cellular level (i.e., Platelet Activating Factor (PAF); 1-O-alkyl-2-acetyl-sn-glyceryl-3-phosphorylcholine) [133]. The analysis of the composition of lipids of nudibranch species (*Chromodoris tinctoria, C. michaeli, C. geometrica, Chromodoris *sp.*, Glossodoris cincta, G. atromarginata, Risbecia tryoni,* and *Platydoris* sp.) showed that the major lipid class was phospholipids with a concentration range from 73.8% in *Chromodoris geometrica* to 81.7% in *Glossodoris cincta* of the total lipids [129]. In a comparable study, lipid analysis was conducted on two nudibranch *molluscs, Chromodoris* sp. and *Phyllidia coelestis*, and phospholipids were the dominating lipid class (85.7 and 54.9% of the total lipids) [134]. The principal phospholipids from the bivalve mollusc *Anadara broughtonii* have been discovered to be phosphatidylcholine and phosphatidylethanolamine (PEA), with their total content of 45.0–54.0% of the phospholipids mass. A similar conclusion was drawn from phospholipids from Mytilus bivalve molluscs, (i.e., *M. edulis* and *M. galloprovincialis*) with a total content of 36.8–43.1% and 25.3–38.5% for phosphatidylcholine and phosphatidylethanolamine, respectively [135].

Marine sterols are one of the lipids that are often found in molluscan species including *Chromodoris tinctoria*, *C. michaeli*, *C. geometrica*, *Chromodoris* sp., *Glossodoris cincta*, *G. atromarginata*,* Risbecia tryoni*, and *Platydoris* sp. [129]. Due to their anti-inflammatory and antioxidant characteristics, marine sterols such as fucosterol and saringasterol have been studied for several health benefits, including anti-cancer, anti-obesity, anti-diabetes, anti-aging, and anti-Alzheimer’s effects [136]. The content of sterols in *Chromodoris tinctoria*, *C. michaeli*, *C. geometrica*, *Chromodoris* sp., *Glossodoris cincta*, *G. atromarginata*, *Risbecia tryoni*, and *Platydoris* sp. ranged from 13.5% to 16.1% of the total lipids, and 13% of the total lipids for *Chromodoris* sp. and *Phyllidia coelestis* [129,134].

Almost all marine molluscs, especially fatty fish like squid and octopuses, are excellent sources of omega-3 polyunsaturated fatty acids (PUFA), such as eicosapentaenoic acid (EPA) and docosahexaenoic acid (DHA) [137]. These fatty acids are known to have cardiovascular benefits in reducing the risk of heart disease, and neurodevelopment abilities in promoting brain health, and they also possess anti-inflammatory properties. They are currently used in the industry as nutraceuticals (fish oil and capsules), for the fortification of livestock, feed, and infant formula [124]. Due to the presence of polyunsaturated fatty acids (PUFA)/ lipinol, the bivalve molluscan *Perna canaliculus* has anti-inflammatory and anti-arthritis properties [125]. However, their fatty acid content is usually low compared to that of their phospholipids and sterols. Moreover, the storage components of the cells, triacylglycerols, and monoalkyl diacylglycerols are usually minor components, ranging between 2.6% and 3.4% of the total lipids [129].

### 5.4. Polyphenolic Compounds Pigments 

Marine polyphenols are found in a variety of natural sources, including molluscs. Shellfish, such as shrimps, clams, and oysters, are a source of marine polyphenols and other minor nutrients [138]. The most common compounds found in shellfish are carotenoids such as astaxanthin and zeaxanthin, which have antioxidant and anti-inflammatory properties [139]. These polyphenols are derived from algae and other marine organisms that are consumed by shellfish as part of their diet [140]. One example of a marine polyphenol is the catechins, which are also found in tea, and procyanidins, which are found in various fruits, vegetables, and brown seaweeds [141]. These polyphenols are believed to have a range of health benefits, including antioxidant and anti-inflammatory effects [138]. 

### 5.5. Marine Enzymes 

Serine and cysteine protease enzymes are found in molluscs are they are used to prevent unwanted colour changes in food products, in meat tenderizing, in the curing of herring, and in squid fermentation [124]. The discovery of the molluscan enzymes is currently underway. The fucoidanase enzyme was isolated and purified from the digestive glands of the marine mollusk *Lambis* sp. [142]. Moreover, the *Nacella concinna* molluscan species produced proteolytic (keratinolytic) and glycolytic (α-L-rhamnosidases) enzymes [143]. The bacterial isolates (GS 1-4, GS 2-1, and GS 2-12) isolated from gastropod species, *namely Conus ebraeus *L.1758 and *Morula aspera*, as well as one bivalve species, *Hiatula chinensis,* showed potent enzymatic activity [144].

### 5.6. Minerals and Vitamins 

Almost all molluscan species (such as oysters and mussels) are often high in essential minerals such as calcium, iron, zinc, selenium, copper, manganese, and iodine and water-soluble vitamins, including B vitamins (B12, B6, and riboflavin), vitamin D, and vitamin A [117]. The minerals are crucial for bone health, immune function, antioxidant defence, and thyroid regulation, and vitamins play important roles in energy metabolism, immune support, vision, and overall health, and they are currently used in food, pharma, and nutraceutical industries [124]. The *Bivalve* class is mostly rich in calcium, iron, zinc, and phosphorus, making it applicable as a food supplement [125].

## 6. Medicinal Importance of Marine Molluscs

The pharmaceutical sector is expanding quickly and consistently [145]. Despite the massive number of pharmaceuticals that are generated annually, the need for new drug discovery remains critical [146]. The emergence of new diseases and infections, the appearance of new challenges in old diseases like AIDS, the rise of drug-resistant infectious diseases, and the highly toxic nature of some currently used drugs are some of the factors driving the search for new medications [21]. Many molluscan species are used in traditional Chinese, Indian, South African, and Middle Eastern medicines, as well as in homeopathic remedies [18,118,120]. Traditional medicines have included molluscan shells, soft tissues, basal portions, mucilage, and even complete molluscan organisms for treating cancer, inflammations, dotage, and other ailments [11]. Diverse marine molluscs have yielded many bioactive compounds which in turn are the driving force towards bioprospecting and drug discovery to reveal their potential to produce novel bioactive compounds with pharmaceutical applications. Researchers have found compounds with potential medicinal properties in the venoms and secretions of various mollusc species that show promise as anti-microbial (antibacterial, antiviral, antifungal), anti-inflammatory, antioxidant, and anti-cancer agents [147]. 

### 6.1. Anti-Microbial Properties

In recent decades, attempts have been made to produce anti-bacterial and immunological drugs to treat and prevent several infectious diseases that affect humans and are caused by germs [148]. The decades of research have shown that marine organisms offer tremendous opportunities to harvest anti-microbial substances as well as provide cues for their laboratory synthesis [147]. Antimicrobial compounds have been generated by marine molluscs like snails, clams, and mussels as defences against infections in their aquatic surroundings. These compounds have antimicrobial effects on viruses, fungi, and bacteria. There are several basic mechanisms that many of these antimicrobial agents have, yet the modes of action can vary depending on the structure of the compound. The antimicrobial compounds are responsible for Disrupting the integrity of microbial cell membranes, creating pores, or causing the leakage of essential components, leading to cell death. Examples include the Myticin and Mytilin from marine mussels, which operate through this mechanism [149].

Inhibiting key enzymes like DNA gyrase and RNA polymerase, disrupting bacterial replication and transcription. These enzymes are essential for microbial growth and survival and may be involved in metabolic pathways or cell wall synthesis [150]. Examples include conotoxins targeting ion channels and neurotransmitter receptors in nerve cells and Aplisynin, a compound isolated from sea hare *Aplysia kurodai* [151].

Causing oxidative stress in bacteria by generating reactive oxygen species (ROS), damaging cellular components like DNA, proteins, and lipids. Marine molluscs like mussels produce molecules like superoxide dismutase or metal-binding proteins that facilitate ROS production such as Mytimycin C which can induce oxidative stress in bacteria [152].

**Figure 3:**
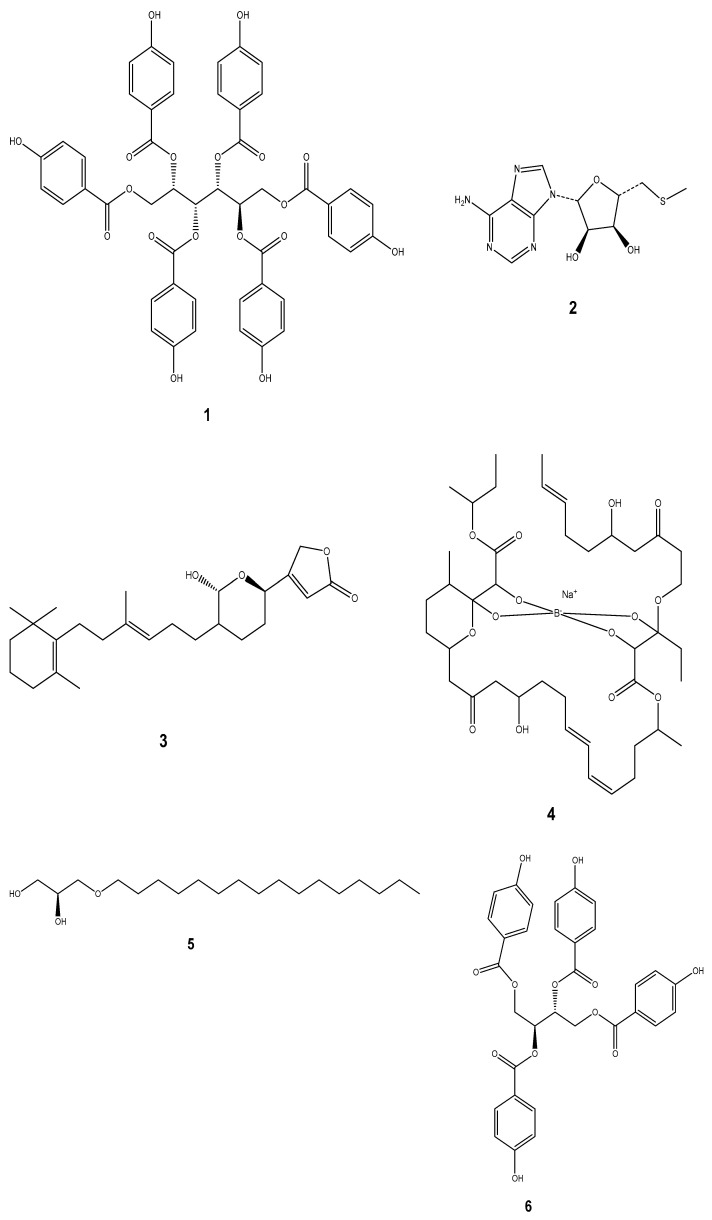

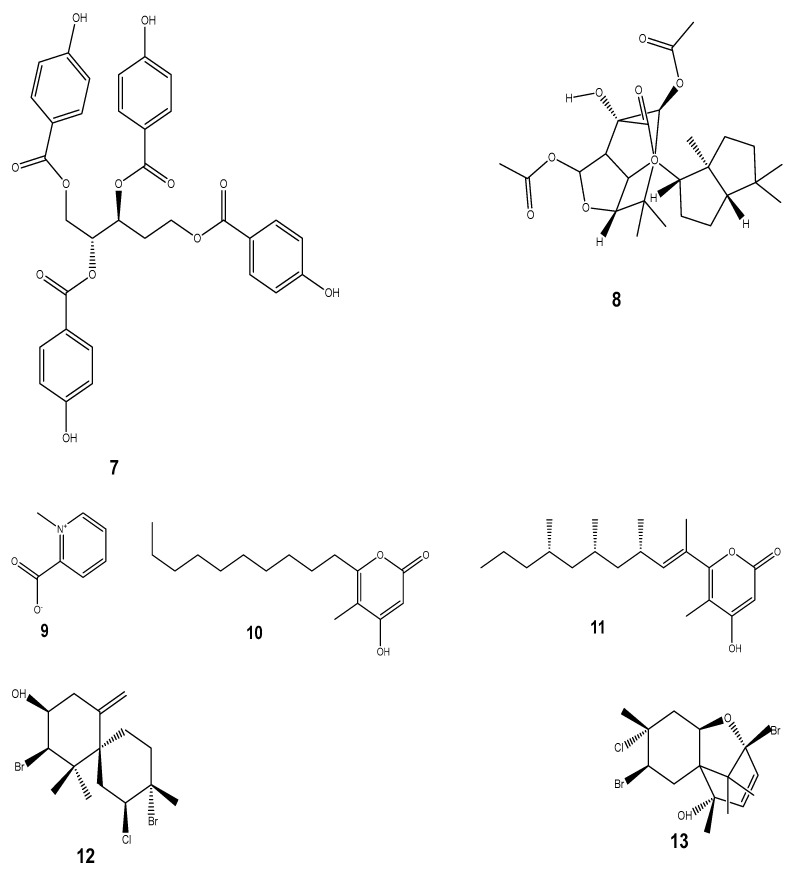
Some compounds isolated from marine molluscs possessing antimicrobial properties.

Marine molluscs have yielded several compounds with anti-microbial properties, including glycoproteins, peptides, indole alkaloids, and chlorinated acetylenes [20]. Among these compounds, we have Scutinin A (**1**) isolated from the Australian limpet *Scutus antipodes*, 5’-deoxy-5’-methylthio-adenosine (MTA) (**2**) from a Dorid nudibranch [153], and a deoxy analog of manoalide (**3**) from *Chromodoris willani* [20]. We also have Tartrolon E (**4**) obtained from a shipworm [153], Hexadecylglycerol (**5**) from *Archidoris montereyensis*, Kelletinin-I and II (**6,7**) from *Kelletia kelletii* [154], and Chromodorolide-A (**8**) from the nudibranch *Chromodoris cavae* [155]. More compounds like Homarine (**9**) from *Marionia blainvillea* and *Aglaja tricolorata*, Diemenensins A (**10**) from *Siphonaria* spp. and *Siphonaria diemenensis*, Pectinatone (**11**) from *Siphonaria pectinate* [13], iso-obtusol (**12**) from *Aplysia Parvula,* and Pacifenol (**13**) from *A. dactylomela* have been isolated [156]. Anti-microbial peptides (AMPs) represent the most universal immune effectors and they are divided into four families, which are defensins, myticins, mytilins, and mytimycins. Several *Bivalves* including *Mytilus galloprovincialis, M. edulis, M. trossolus, Crassostrea virginica, Ruditapes philippinarum,* and *Gastropods* like *Biomphalaria glabrata, Haliotis discus hannai, H. discus discus,* and *H. laevigata* form important sources of AMPs. This entails that so far, AMPs have been isolated only from these two major groups of molluscs (Figure 3) [147].

### 6.2. Anti-Cancer Properties

**Figure 4:**
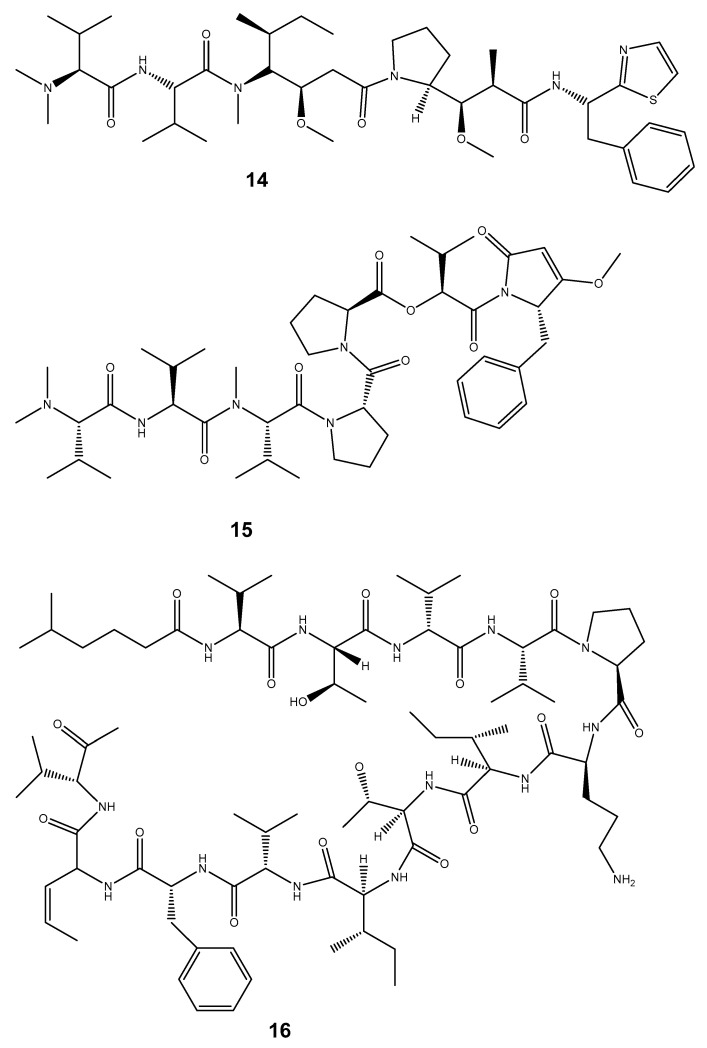

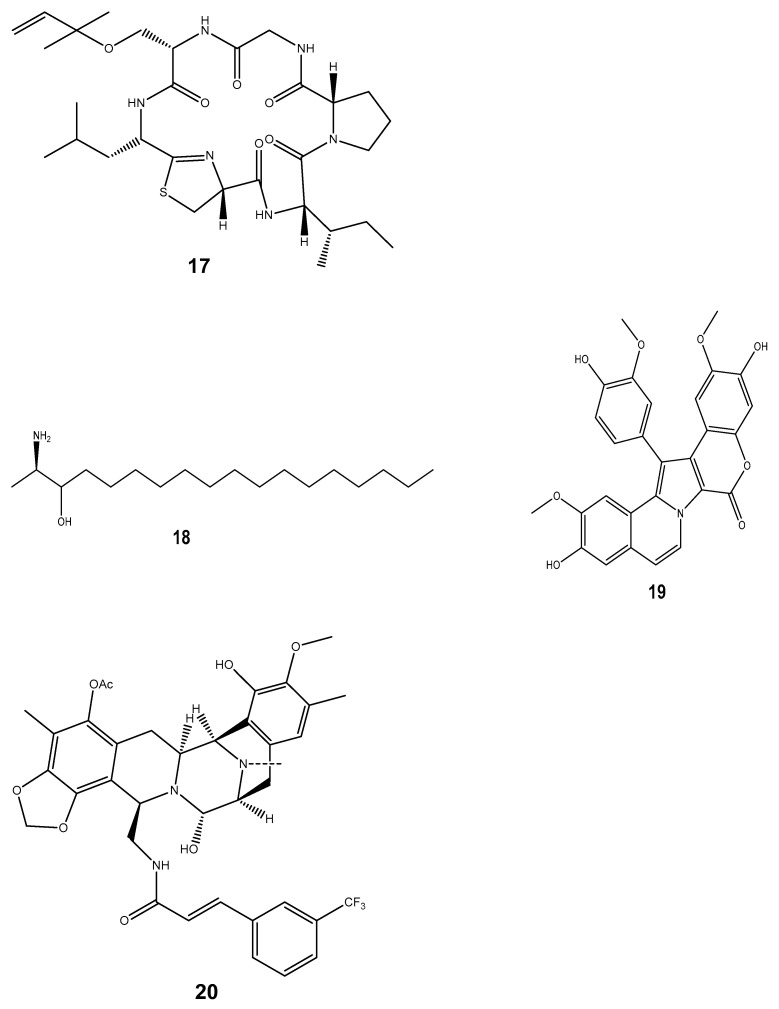

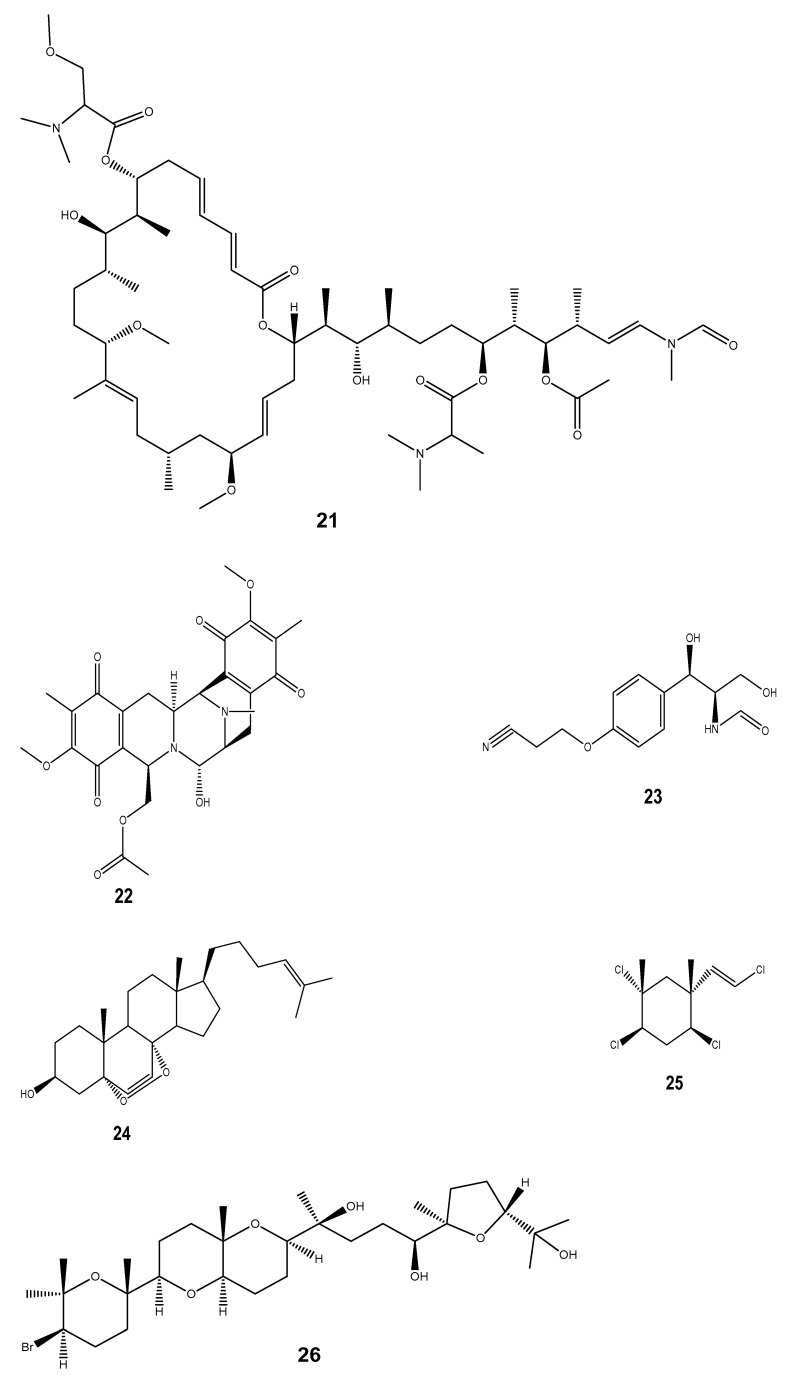
Some compounds isolated from marine molluscs possessing anticancer properties.

Oceans are now considered a treasure trove of bioactive compounds possessing anti-cancer (antioxidant/cytotoxicity/anti-tumour) activities [147]. According to the Food and Drug Administration (FDA) and the European Agency for the Evaluation of Medicinal Products, among the newly discovered metabolites with promising anti-tumour properties, some are from the marine environment [157,158]. However, the marine bioactive compounds constitute a small percentage, which suggests the need for more effort to discover novel anti-tumour compounds [147]. Chemical investigations of the phylum Mollusca have described various compounds as potential anticancer drugs based on their ability to overcome cancer cell resistance chemotherapy. The reason behind the selection of mollusc-derived anticancer drug candidates was due to their ability to target the biological characteristics of cancer cells, and their potency, selectivity, and mechanisms of action along with their alimentary behaviour [20]. Molluscan compounds including peptides were found to possess anticancer properties through different mechanisms of action in the killing of cancer cells such as apoptosis induction, cell cycle arrest, angiogenesis inhibition, and metastasis inhibition. Some of the bioactive compounds possessing anti-tumour/anticancer properties from marine molluscs include the linear peptide Dolastatin 10 (**14**) and desipeptide Dolastatin 15 (**15**) isolated from *Dollabella Auricularia* and Kahalalide F (**16**) from *Elysia rufescens* [147]. The Keenamide A (**17**) obtained from *Pleurobranchus forskalii* has also shown these properties, as well as the alkyl amino alcoholic compound Spisulosine ES-285 (**18**) from the arctic surf clam *Spisula polynyma,* and Lamellarin D (**19**) from *Lamellaria* [147]. Other compounds have similarly been reported to possess these activities, like Zalypsis (**20**) isolated from the pacific nudibranch mollusc *Joruna funebris*, Aplyronine A (**21**) from *Aplysia kurodai* [20], Jorumycin (**22**) from the doridacean nudibranch *Jorunna funebris* and Bursatellanin (**23**) from *Bursatella leachii* [13]. We also noted 5α,8α-epidioxysterols (**24**) isolated from *Aplysia punctate*, the cyclic monoterpene aplysia terpenoid A (**25**) from *Aplysia kurodai,* and Thyrsiferol (**26**) from *Laurencia thyrsifera* [156], which exhibited this activity (Figure 4).

### 6.3. Anti-Inflammatory and Analgesic Properties

**Figure 5:**
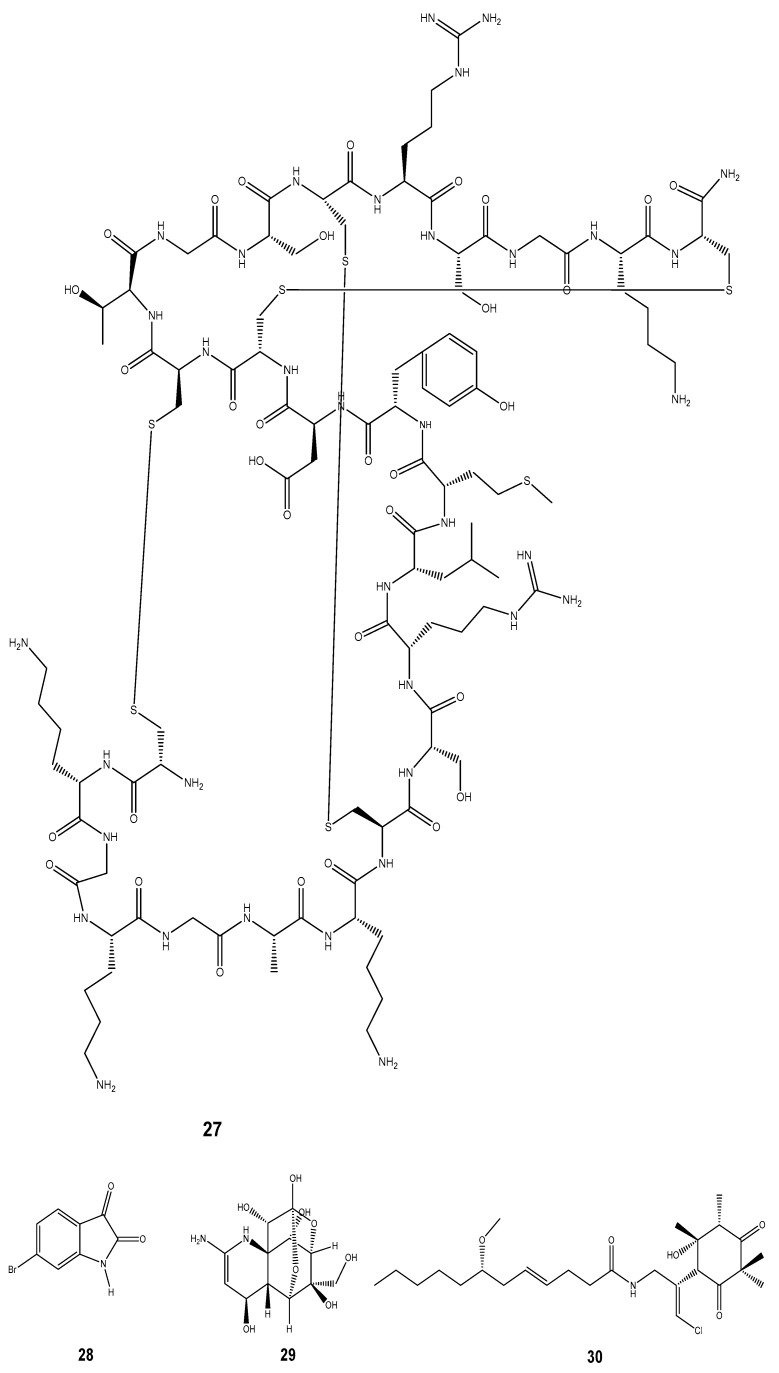

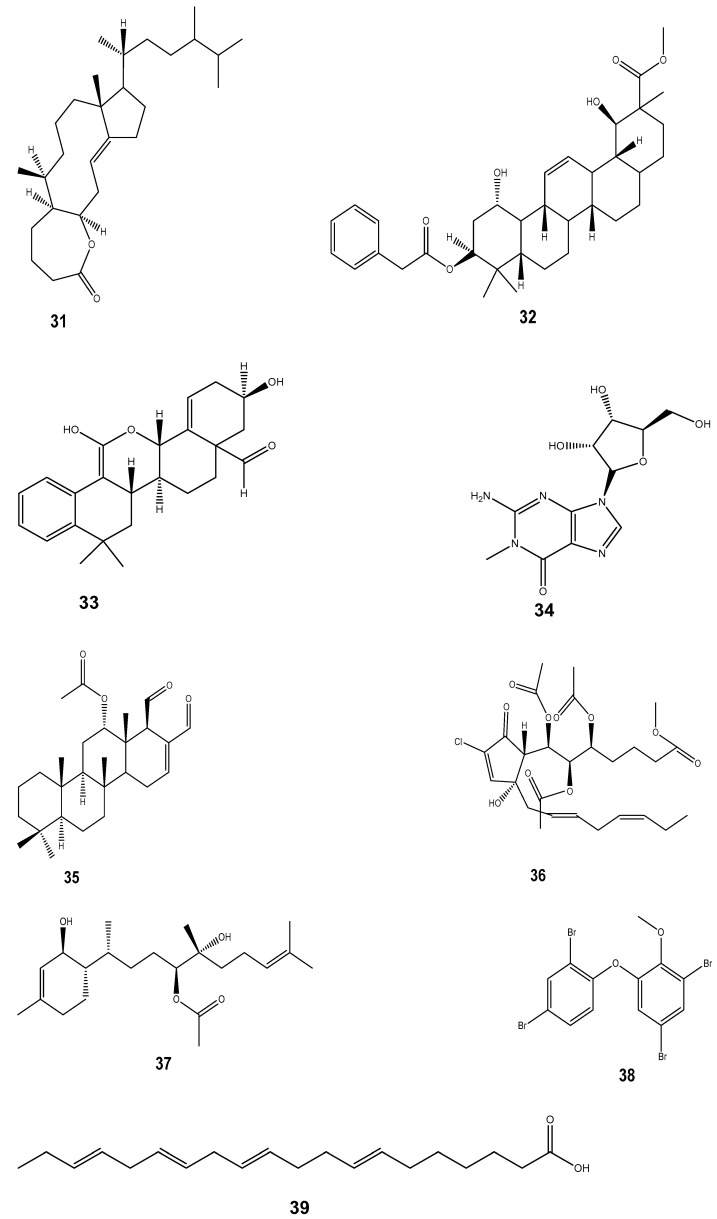
Some compounds isolated from marine molluscs possessing anti-inflammatory and analgesic properties.

Inflammation is the complex biological response of the vascular system which arises as the product of oxidative stress [159]. It is usually associated with pains and other sufferings requiring immediate medication. Great progress has been made in recent decades towards understanding the neural substrates of pain relief and identifying novel molecular targets for anti-inflammatory and analgesic drug development [160]. A series of bioactive compounds with promising anti-inflammatory and analgesic properties have been identified and isolated from marine molluscs [147]. Peptides and proteins having analgesic and anti-inflammatory characteristics are a frequent mechanism of action for these properties [161]. Conotoxins are one prominent case in point. Conus snails’ (genus *Conus*) venom contains tiny peptides known as conotoxins [14]. These toxins are used by cone snails, a type of marine mollusc, to paralyze their prey [162]. In the neurological system, conotoxins can interact with ion channels and receptors. Some of them target nicotinic acetylcholine receptors, calcium channels, or voltage-gated sodium channels [163]. Conotoxins can interfere with pain signals and lessen inflammation by blocking or altering the activation of these channels [147]. Several reported marine molluscs have bioactive compounds possessing anti-inflammatory/ analgesic properties including the Ziconotide (**27**) isolated from *Conus geographus and Conus magus* [147], 6-bromoisatin (**28**) from the Australian marine mollusc *Dicathais orbita* [20], and tetrodotoxins (**29**) from bivalves and gastropod samples [164]. We also noted the Malyngamide S derivative (**30**) obtained from *Bursatella leachii*, the lactonic disecosteroid 9-disecoergosta-8-en-α-homo-6a-oxa-1-one (**31**) from *Babylonia spirata*, the phenylacetyloxy-trimethylpicene-23-carboxylate derivative (**32**) from *Crassostrea madrasensis,* and the benzo[h]naphtho[1,2-c] chromene derivative (**33**) from *Perna viridis* [20]. Other compounds exhibited this analgesic property, among which was 1-methyl-isoguanosine (**34**) isolated from the nudibranch *Anisodoris nobilis* [154], Scalaradial (**35**) from *Glossodoris pallida*, Punaglandin (**36**) from *Tritonia* sp. and Dactyloditerpenol acetate (**37**) from *Aplysia dactylomela* [13]. A polybrominated diphenyl ether (**38**) isolated from *Aplysia dactylomela* [156], and 17-eicosatetraenoic acid (**39**) from *Perna canaliculus* have also been reported [165] (Figure 5).

### 6.4. Antioxidant Properties

**Figure 6:**
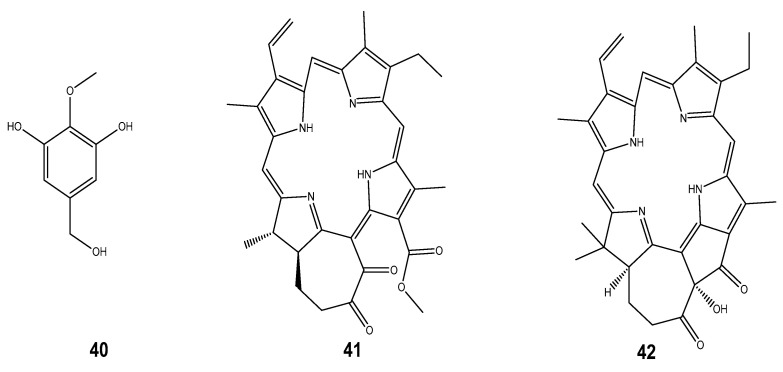

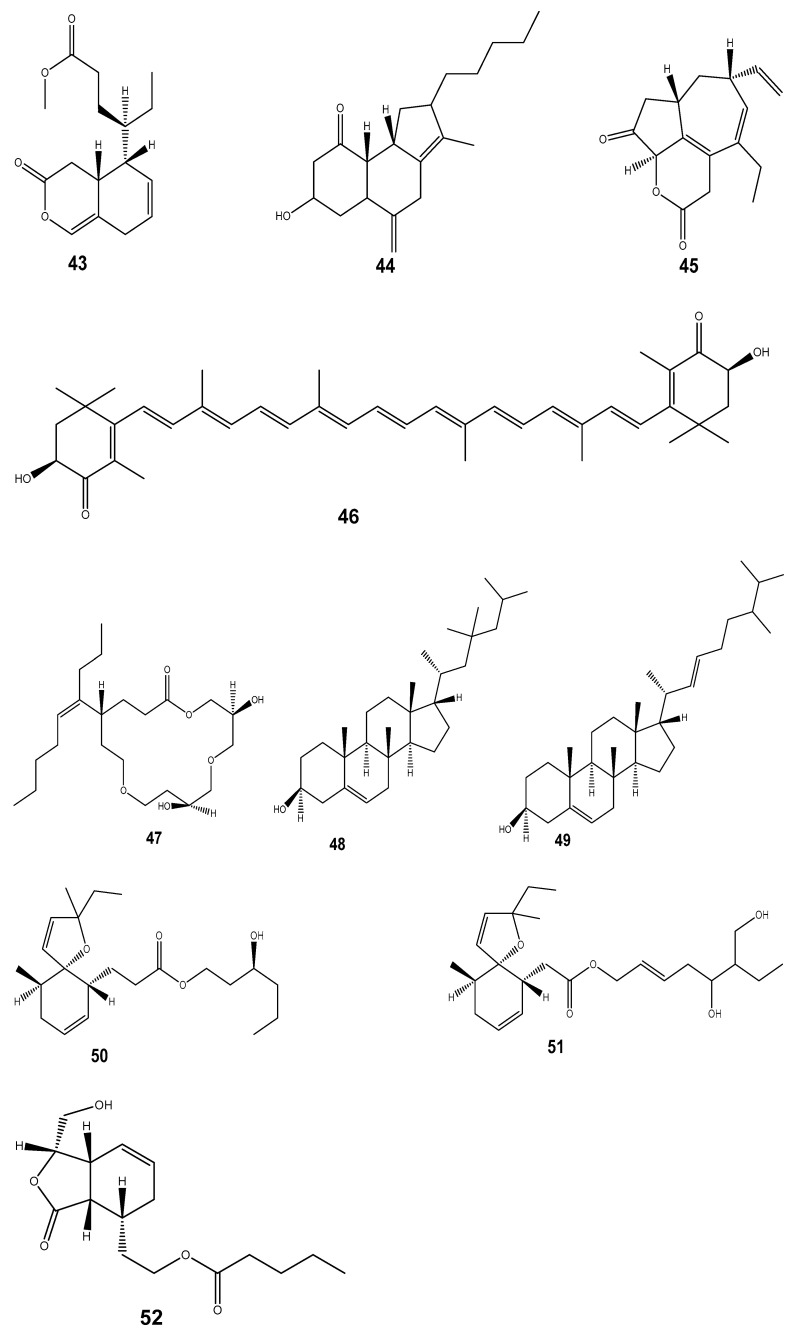
Some compounds isolated from marine molluscs possessing antioxidant properties.

The excessive reactive oxygen species (ROS) (including hydroxyl radical (^•^OH), superoxide anion (O_2_^•-^), hydrogen peroxide (H_2_O_2_), nitroxide radicals (NO^•^), and peroxyl radicals (ROO^-^)) results in oxidative stress which can cause the pathogenesis of various chronic diseases such as atherosclerosis, diabetes, cancer, arthritis, and the ageing process [166]. In the past few years, mollusc-derived antioxidants (including taurine, carotenoids, α-tocopherol and n-3 polyunsaturated fatty acids, polysaccharides, and peptides), especially those from bivalve and gastropod groups of molluscs, were discovered [83]. The biological pathways of these natural antioxidants are either unknown or not well understood; however, n-3 polyunsaturated fatty acids used as a dietary supplement have been known to mitigate oxidative stress through the induction of cellular antioxidant responses [83]. These compounds could alleviate oxidative stress-mediated diseases by scavenging free radicals. This, in turn, relieves the cellular damage caused by oxidation, and they have been added to health supplements, food additives, and pharmaceuticals [167]. On the other hand, polyphenols are well-known, strong antioxidants due to their common mechanism of donating hydrogen atoms or electrons to neutralize free radicals the effect could be explained by three distinct mechanisms, including scavenging the ROS, regulating the antioxidant system, or oxidative stress-mediated signaling pathways [166]. Some compounds exhibiting antioxidant properties include 3,5-dihydroxy-4-methoxybenzyl alcohol (**40**) isolated from *Crassostrea gigas* [83], and Chlorophyllonic acid A methyl ester (**41**) and Chlorophyllone A (**42**) from *Ruditapes philippinarum* [20]. Chromenyl derivative methyl 9-(tetrahydro-3-oxo-3H-isochromen-5-yl) hexanoate (**43**) obtained from the spineless cuttlefish *Sepiella inermis* also exhibited this activity as well as Ramosane (**44**) from *Chicoreus ramosus*, Octahydroazulenopyrandione (**45**) from *Amphioctopus marginatus,* and Astaxanthin (**46**) from Octopus and cuttlefish species [20]. We also noted that polyether macrocyclic lactone (**47**) isolated from *Babylonia spirata* [168], 23-gem-dimethylcholestaenol and methyldihomocholest-5, 22-dienol (**48, 49**) from *Paphia malabarica* [169], and O-spirocyclic ether derivatives and irregular meroterpenoid derivatives (**50, 51, 52**) from *Villorita cyprinoides* have been reported for their activities [170] (Figure 6).

The presence of certain functional groups in the compound determines its affinity for specific receptors and enzymes, thereby influencing its pharmacological activity and therapeutic effects [171,172]. Some compounds have shown high potency and have been successful in reaching clinical trials and the pharmaceutical market, as shown in Table 2.

## 7. Limitations, Gaps, and New Perspectives

Taking consideration of the biodiversity of the molluscan species, there is currently limited research on the extraction of bioactive compounds from them. Even though some marine molluscs have been the subject of substantial research due to their nutritional and therapeutic value, such as certain species of gastropods, bivalves, and cephalopods, many others have received comparatively little attention. We are unable to fully comprehend the range of health benefits and bioactive compounds found in various molluscan species due to a dearth of studies. In addition, the utilization of non-conventional extraction techniques in extracting bioactive compounds from marine molluscs remains relatively understudied compared to conventional methods. Non-conventional extraction techniques often involve a range of parameters, such as frequency and power for ultrasound-assisted extraction or temperature and pressure for supercritical fluid extraction, which can influence extraction efficiency and compound yield. There is limited research on the mode of action of discovered molluscan bioactive compounds. The study of the mode of action of bioactive compounds helps researchers to

(1) Understand how these compounds exert their effects on biological systems, whether it is by targeting specific molecular pathways, receptors, enzymes, or other mechanisms;
(2) Identify potential therapeutic targets for drug development. This knowledge can lead to the creation of new drugs or therapeutic interventions that have specific effects on biological processes;
(3) Optimize treatment regimens, dosages, and combinations. This knowledge can help improve the efficacy of treatments and reduce potential side effects.

Furthermore, incorporating cultural and indigenous knowledge into scientific research can improve molluscan research, as since ancient times, coastal communities and indigenous populations have relied on marine molluscs for food and medicine. They also frequently have traditional knowledge of the benefits of these molluscs. Scientific research can benefit from the inclusion of this indigenous knowledge to foster culturally aware methods of using molluscs and gain important insights into their potential as medicines. Ultimately, resolving these limitations and investigating fresh angles in the research on the medicinal and nutraceutical significance of marine molluscs will enhance our understanding of these intriguing organisms and unlock their full potential for promoting human health and well-being. 

## 8. Conclusions and Outlooks

This review gives us a broad spectrum of marine molluscs, their means of extraction and crucial components, as well as their being vital sources for human nutrition and medicine. They are subjects of continuous research and economic interest due to their wide variety of bioactive compounds and nutritional benefits, emphasizing their significance in both ecological and human contexts. Further research on their medicinal and nutraceutical potential could improve people’s health and advance medical science. Additionally, further investigation is required to completely comprehend their modes of action, recommended consumption patterns, and potential adverse effects.

## Figures and Tables

**Table 2:** Therapeutic effects and modes of action of notable market-approved mollusc-derived compounds.

Compound	Mollusc Species	Therapeatic Effect	Associated Company/institution	Mode of Action	References
Ziconotide (ω-conotoxin) (**27**)	*Conus geographus* and *Conus magus*	Anti-inflammatory and analgesic	Elan corporation	Disrupts the calcium channel at the neuromuscular junction that is involved in the transmission of nerve impulses. The pain sensitivity is associated with calcium channels.	[173]
Dolastatin 10 (**14**)	*Dollabella auricularia*	Anti-cancer	Celltrion pharmaceutical company	Interferes with and hinders mitotic cell division. Due to its potent capacity to block the mitotic cell cycle, it may be able to specifically target cancer cells.	[174,175]
Kahalalide-F (**16**)	*Elysia rufescens*	Anti-cancer	Pharmamar	Induces oncosis in cancerous cells via the lysosomal induction and permeabilization of the cell membrane. Furthermore, the compound also suppresses the expression of genes involved in DNA replication and cell proliferation, which may prevent tumour development and spread.	[175]
